# Endoscopic third ventriculostomy for diencephalosynapsis-related hydrocephalus in MPDZ gene syndrome: a case report and review of the literature

**DOI:** 10.1007/s00381-026-07483-7

**Published:** 2026-10-02

**Authors:** Sergio Cavalheiro, Marcos Devanir Silva da Costa, Fernando Seiji Suzuki, Patrícia Alessandra Dastoli, Diego Pereira de Melo Oliveira, Rocio Ester Gómez Verges, Gabriela Cavalieri de Oliveira, Mariana Rodrigues Martino

**Affiliations:** Department of Neurology and Neurosurgery, Universidade Federal de São Paulo (UNIFESP) , Rua Napoleão de Barros, 715, 6Th Floor, São Paulo, SP 04024-002 Brazil

**Keywords:** MPDZ syndrome, Hydrocephalus, Endoscopic third ventriculostomy, Diencephalosynapsis

## Abstract

**Purpose:**

MPDZ syndrome is a rare autosomal recessive disorder caused by biallelic pathogenic variants in MPDZ (9p23) and typically managed with ventriculoperitoneal shunting (VPS). Recent characterization of MPDZ-related hydrocephalus as an obstructive process—driven by diencephalosynapsis and third ventricle atresia—provides theoretical grounds for endoscopic third ventriculostomy (ETV), yet no primary ETV treatment has previously been reported in a genetically confirmed, liveborn patient.

**Methods:**

We report a female infant with prenatally diagnosed obstructive ventriculomegaly, subsequently confirmed as MPDZ syndrome by whole-exome sequencing, who was treated with ETV as the primary surgical intervention, and we review the literature on the neurosurgical management of MPDZ-related hydrocephalus.

**Results:**

Fetal MRI demonstrated diencephalosynapsis and third ventricle atresia with secondary occlusion of the aqueduct of Sylvius. A ventriculo-amniotic shunt was placed at 29 weeks of gestation; the infant was born at 36 weeks and 1 day and underwent ETV on the fifth day of life without complications. At 2 years and 5 months of age, she developed intermittent symptoms of intracranial hypertension due to late stoma occlusion at the level of the Liliequist membrane, successfully managed with endoscopic stoma revision. At the most recent follow-up (4 years and 5 months), she remains free of ventriculoperitoneal shunting, with age-appropriate neurodevelopment. A systematic review of the literature identified no prior report of ETV used as primary treatment in a genetically confirmed, liveborn patient with MPDZ syndrome.

**Conclusions:**

This case provides proof of concept that ETV is both mechanistically justified and clinically effective in selected patients with MPDZ-related obstructive hydrocephalus, potentially avoiding lifelong shunt dependency. Patient selection should nonetheless remain individualized, based on ventricular anatomy, third ventricular floor thickness, and associated structural anomalies.

## Introduction

MPDZ syndrome is a rare genetic condition characterized by severe congenital hydrocephalus (CH), inherited in an autosomal recessive pattern. It results from pathogenic variants in the MPDZ gene on chromosome 9p23, which encodes a protein essential for tight-junction integrity [1]^.^ Within the central nervous system, MPDZ localizes predominantly to the ependyma and choroid plexus, where it plays a critical role in maintaining the blood–brain barrier [4, 20, 28].

The underlying pathophysiology involves early ependymal denudation followed by reactive astrogliosis [4, 20], a process that frequently produces aqueductal stenosis and obstructive hydrocephalus [1, 3, 4]. Recent studies have shown that pathogenic MPDZ variants produce a characteristic obstructive pattern related to diencephalosynapsis, defined by partial fusion of the thalami and third ventricle atresia [3].

Although initially described as non-syndromic hydrocephalus [1], the phenotypic spectrum has since expanded considerably [23, 24].

Systemic manifestations include severe ocular anomalies as well as complex cardiac malformations, renal anomalies, and Dandy–Walker malformation [13, 23, 24]. Clinical variability is striking, ranging from prenatally fatal presentations to mild, non-progressive communicating hydrocephalus [2, 3]. Recognition of specific anatomical clues is essential for accurate genetic diagnosis, appropriate clinical management, and reproductive counseling [3, 6]. The case reviewed below illustrates this syndrome, correlating the neuroradiological findings of obstructive hydrocephalus with the pathogenic MPDZ variant identified and demonstrating the feasibility of early treatment with third ventriculostomy, contributing to the growing body of evidence on this rare disorder [3, 19, 20].

## Historical background

Hydrocephalus has traditionally been defined as active ventricular distension resulting from impaired dynamics of cerebrospinal fluid (CSF) between its sites of production and absorption [17, 30]. Recent genomic reviews, however, have proposed a shift, reclassifying genetic forms of hydrocephalus such as MPDZ syndrome (Congenital Hydrocephalus type 2; MIM: 615219) as disorders of neural progenitor development. This autosomal recessive condition maps to the 9p23 locus and represents a critical failure of the blood–brain barrier [4, 13].

Historically, isolated genetic CH was attributed almost exclusively to the L1CAM gene. In 2013, Al-Dosari et al. identified MPDZ as the first recessive locus for severe CH in humans [1]. Since then, the phenotypic spectrum has broadened from isolated hydrocephalus to a complex multiorgan syndrome. Current evidence documents the association of MPDZ variants with severe ocular anomalies (colobomas and macular dystrophies), complex cardiac malformations, and renal anomalies, underscoring the pleiotropic role of this gene in embryonic development [23, 24].

The MPDZ protein (MUPP1) acts as an essential molecular scaffold for the stability of apical tight junctions [4, 20]. During embryogenesis, MPDZ operates in synergy with CRB2 and CCDC88C (DAPLE) to regulate apical constriction of neuroepithelial cells, a process fundamental to neurulation and formation of the ventricular system [3, 14]. Failure of this molecular complex leads to early neuroependymal denudation and reactive astrogliosis, producing the diagnostic anatomical signatures of diencephalosynapsis (thalamic fusion) and third ventricle atresia that together define the pattern of progressive obstructive hydrocephalus [3].

## Clinical presentation

The clinical presentation of MPDZ syndrome is marked by striking phenotypic heterogeneity, ranging from mild, non-progressive communicating hydrocephalus to severe, prenatally lethal obstructive forms [2, 3, 24]. Congenital hydrocephalus is the cardinal finding, often detected by fetal ultrasonography from around the 20th gestational week and characterized by progressive supratentorial ventriculomegaly, cortical thinning, and the “dangling choroid plexus” sign [1, 3, 20].

Neurologically, recent studies highlight diagnostic anatomical signatures such as diencephalosynapsis (partial thalamic fusion) and third ventricle atresia, which frequently result in aqueductal stenosis and obstruction of CSF flow [3]. The spectrum of central nervous system malformations has expanded to include Dandy–Walker malformation, lissencephaly, corpus callosum agenesis, and periventricular gray-matter nodular heterotopia [1, 3, 20]. Seizures and neuropsychomotor developmental delay are common among survivors, although cases with preserved cognitive development have also been reported [2, 3].

Systemically, the syndrome manifests as a complex multiorgan disorder. The ocular phenotype includes chorioretinal colobomas, bilateral macular dystrophy, and megalocornea. Severe cardiac malformations have been documented, including aortic coarctation, ventricular septal defects, and single-ventricle physiology. Other associated extracerebral manifestations include renal anomalies and diaphragmatic hernia [1, 3, 23, 24]. Clinical variability is observed even among siblings carrying identical pathogenic variants, suggesting the influence of genetic or environmental modifiers on disease expressivity [3, 24].

## Diagnosis

Diagnosis of MPDZ syndrome requires a comprehensive approach integrating fetal ultrasonography, advanced magnetic resonance imaging (MRI), and genomic analysis [3, 4, 6]. Prenatally, initial suspicion is based on the detection of bilateral, symmetric supratentorial ventriculomegaly on ultrasonography, frequently accompanied by cerebral parenchymal thinning. Fetal and postnatal MRI is essential to identify the pathognomonic anatomical signature of the condition—diencephalosynapsis, defined by partial or complete thalamic fusion together with atresia or severe narrowing of the third ventricle [3]. This finding is critical for differential diagnosis, as it differs from the aqueductal stenosis pattern observed in X-linked (L1CAM) hydrocephalus, in which the third ventricle is dilated [3, 6]. Neuroimaging may additionally reveal associated malformations such as Dandy–Walker malformation and periventricular heterotopia [3, 20].

Definitive confirmation relies on molecular genetic analysis, typically whole-exome sequencing (WES) or whole-genome sequencing (WGS), to identify biallelic pathogenic MPDZ (locus 9p23) [1–4, 13]. Recognition of systemic clinical clues, such as chorioretinal colobomas and cardiac anomalies, should raise diagnostic suspicion, particularly in the setting of parental consanguinity [1, 23, 24]. In postmortem neuropathological studies, the diagnosis is corroborated by identification of multifocal ependymal rosettes and primary neuroependymal denudation [4, 20].

## Management

Management of congenital hydrocephalus in MPDZ syndrome is primarily neurosurgical and should be individualized according to the severity of ventriculomegaly and CSF dynamics [1, 2]. Ventriculoperitoneal shunting (VPS) has traditionally been the first-line intervention for controlling intracranial pressure in severe cases [1, 3]. However, the recent characterization of MPDZ syndrome as a form of obstructive hydrocephalus—frequently associated with anatomical signatures such as diencephalosynapsis and partial third ventricle atresia—provides a robust theoretical basis for neuroendoscopic techniques [2, 4].

Endoscopic third ventriculostomy (ETV) has emerged as a promising therapeutic alternative, particularly in patients presenting an obstructive pattern distal to the lateral ventricles [2, 4]. Recent reports document the clinical success of ETV performed early in life (around the fourth month), enabling effective ventricular decompression and reducing dependence on prosthetic hardware [2]. The choice between external diversion and neuroendoscopy should consider the specific ventricular anatomy identified on advanced MRI, bearing in mind that immediate postnatal surgical intervention is the factor most strongly correlated with preservation of neurological function [1, 4]. Beyond hydrocephalus management, multidisciplinary follow-up is mandatory for seizure control and monitoring of associated systemic malformations [2, 5].

## Outcomes and prognosis

The prognosis of patients with MPDZ syndrome is characterized by marked phenotypic heterogeneity, ranging from perinatal lethality to preserved neuropsychomotor development [1, 2, 5, 6]. In the most severe cases—particularly those associated with homozygous null mutations leading to massive ependymal denudation—the outcome is frequently fetal or early infant death due to massive, progressive hydrocephalus [1, 7]. The literature also documents milder clinical forms with non-progressive communicating hydrocephalus, in which patients reach normal developmental milestones without requiring surgical intervention [2, 6].

Among survivors requiring neurosurgical intervention, cognitive prognosis appears directly related to the timing of treatment and the severity of associated cortical malformations, such as lissencephaly and nodular heterotopia [1, 2, 7]. While some survivors show reduced intelligence quotient (IQ 60–80) and seizures, others exhibit normal cognitive development with only mild attentional disturbances [1, 2]. Systemic prognosis is shaped by the pleiotropic nature of the syndrome, being conditioned by the severity of ocular anomalies such as macular coloboma and by cardiac or renal malformations [5, 8, 9]. Understanding that MPDZ syndrome may present as a complex multiorgan disorder is crucial for accurate prognostic counseling of families [5, 10].

## Case report

**Fig. 1:**
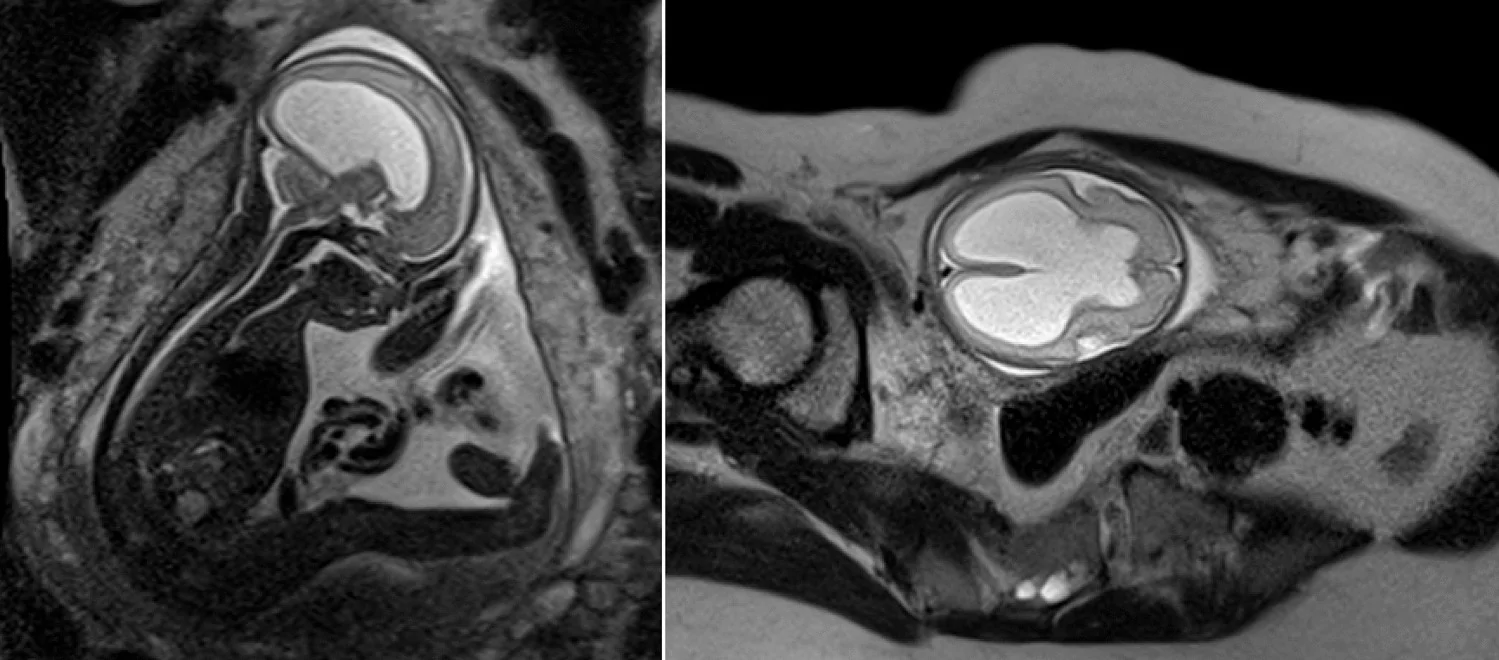
Fetal MRI at 28 weeks of gestation demonstrating marked ventricular dilatation and aqueductal stenosis

A 28-year-old woman, gravida 5 para 4, was referred for fetal MRI after second-trimester ultrasonography raised suspicion of fetal hydrocephalus. Fetal MRI revealed bilateral ventriculomegaly together with hypoplasia of the septum pellucidum and corpus callosum, diencephalosynapsis, and third ventricle atresia with secondary occlusion of the aqueduct of Sylvius—findings consistent with severe obstructive congenital hydrocephalus (Fig. 1).

Fetal intervention with placement of a ventriculo-amniotic shunt was performed at approximately 29 weeks of gestation, without immediate complications. The infant was born prematurely at 36 weeks and 1 day of gestational age by elective cesarean section in cephalic presentation, with Apgar scores of 8 at 1 min and 9 at 5 min. Birth weight was appropriate for gestational age.

At birth, no shunt material was identified over the scalp, and the puncture site was completely healed, suggesting spontaneous extrusion of the ventriculo-amniotic catheter prior to delivery.

During the postnatal period, additional anomalies consistent with the expanded malformation spectrum of MPDZ syndrome were identified, including pyelocaliceal duplication, vesicoureteral reflux, and a hemodynamically significant patent foramen ovale (PFO).

Molecular genetic investigation was initiated, and whole-exome sequencing subsequently confirmed the diagnosis of MPDZ syndrome (OMIM #615219) through identification of a pathogenic variant in the MPDZ gene.

**Fig. 2:**
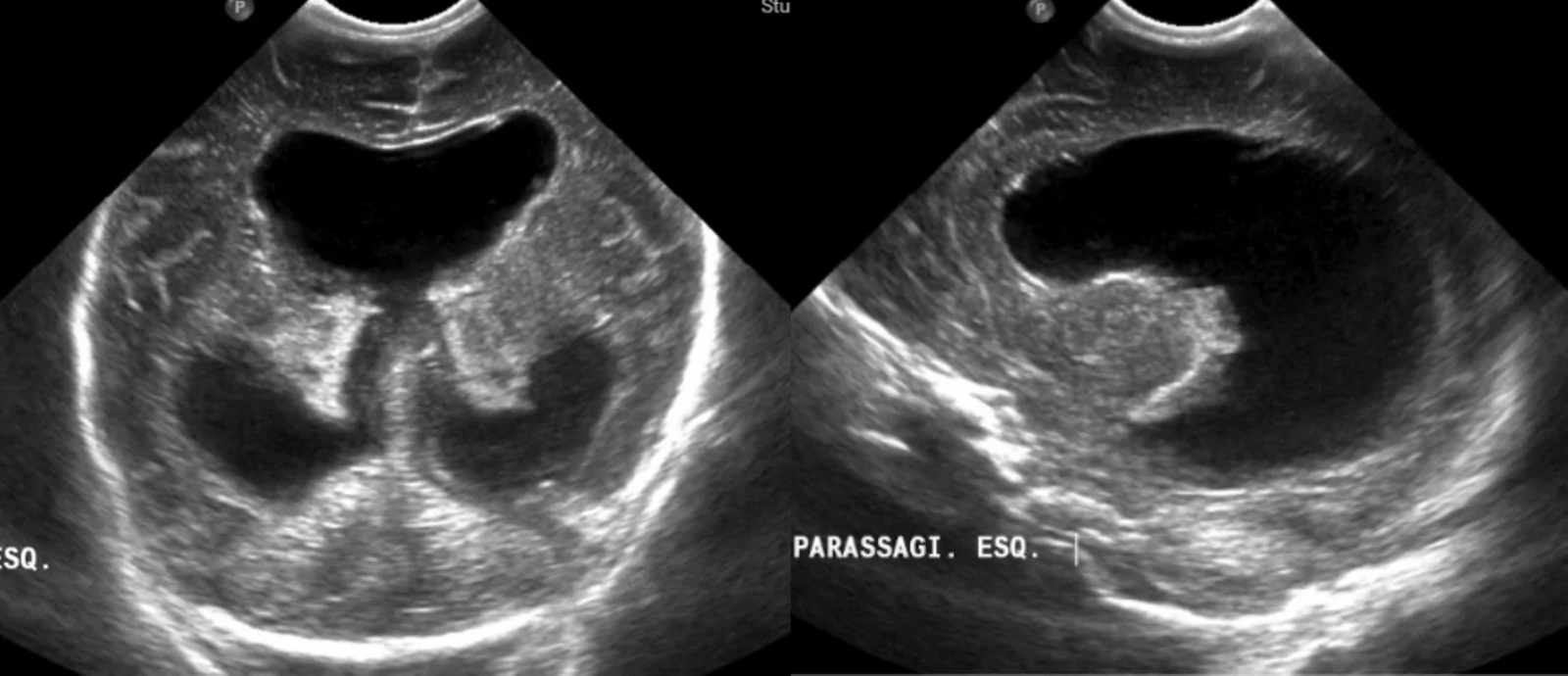
Transfontanelle ultrasonography performed shortly after birth, demonstrating dilatation of the lateral ventricles.

Transfontanellar ultrasonography after birth continued to demonstrate hydrocephalus. On the fifth day of life, the patient therefore underwent endoscopic third ventriculostomy (ETV). The procedure was performed without intraoperative or perioperative complications (Fig. 2).

**Fig. 3:**
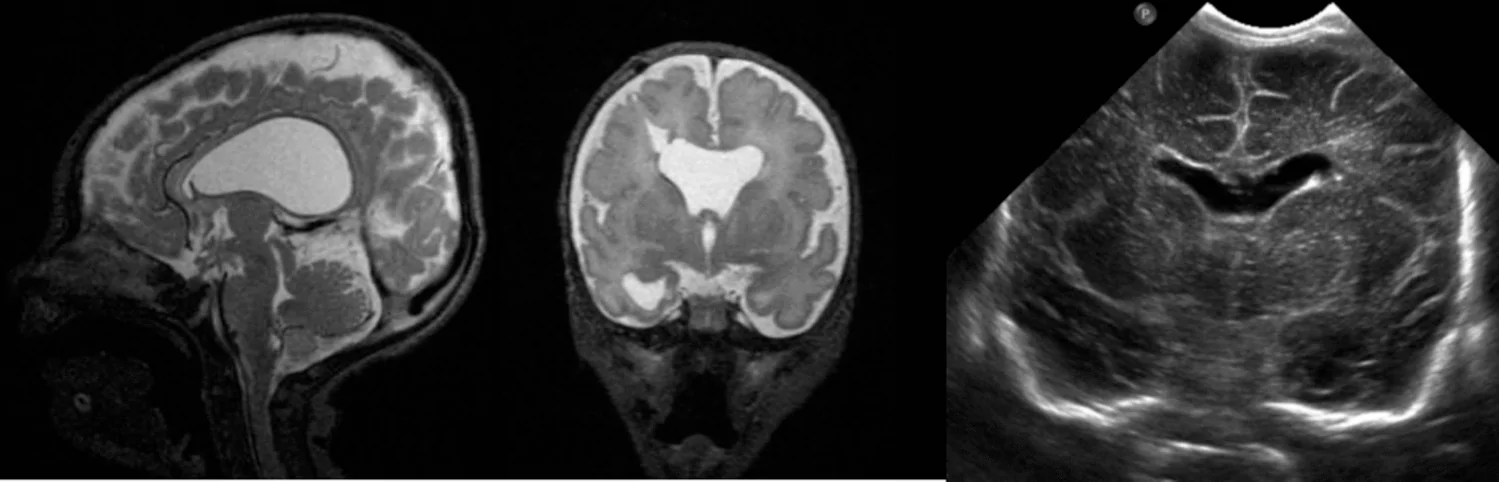
Fetal MRI on postoperative day 4 and transfontanellar ultrasonography on postoperative day 12, demonstrating improvement of ventriculomegaly

Fenestration of the third ventricular floor (tuber cinereum) was achieved under direct endoscopic visualization, establishing communication between the ventricular system and the prepontine cistern. Adequate pulsatile CSF flow through the stoma was confirmed (Fig. 3).

At 1 month of age, the patient underwent successful surgical correction of the patent foramen ovale. She was discharged on antibiotic prophylaxis because of the identified genitourinary anomalies.

At 2 years and 5 months of age, the child returned for medical evaluation with a history of progressive somnolence, decreased school activity, episodes of vomiting, pallor, and bradycardia.

**Fig. 4:**
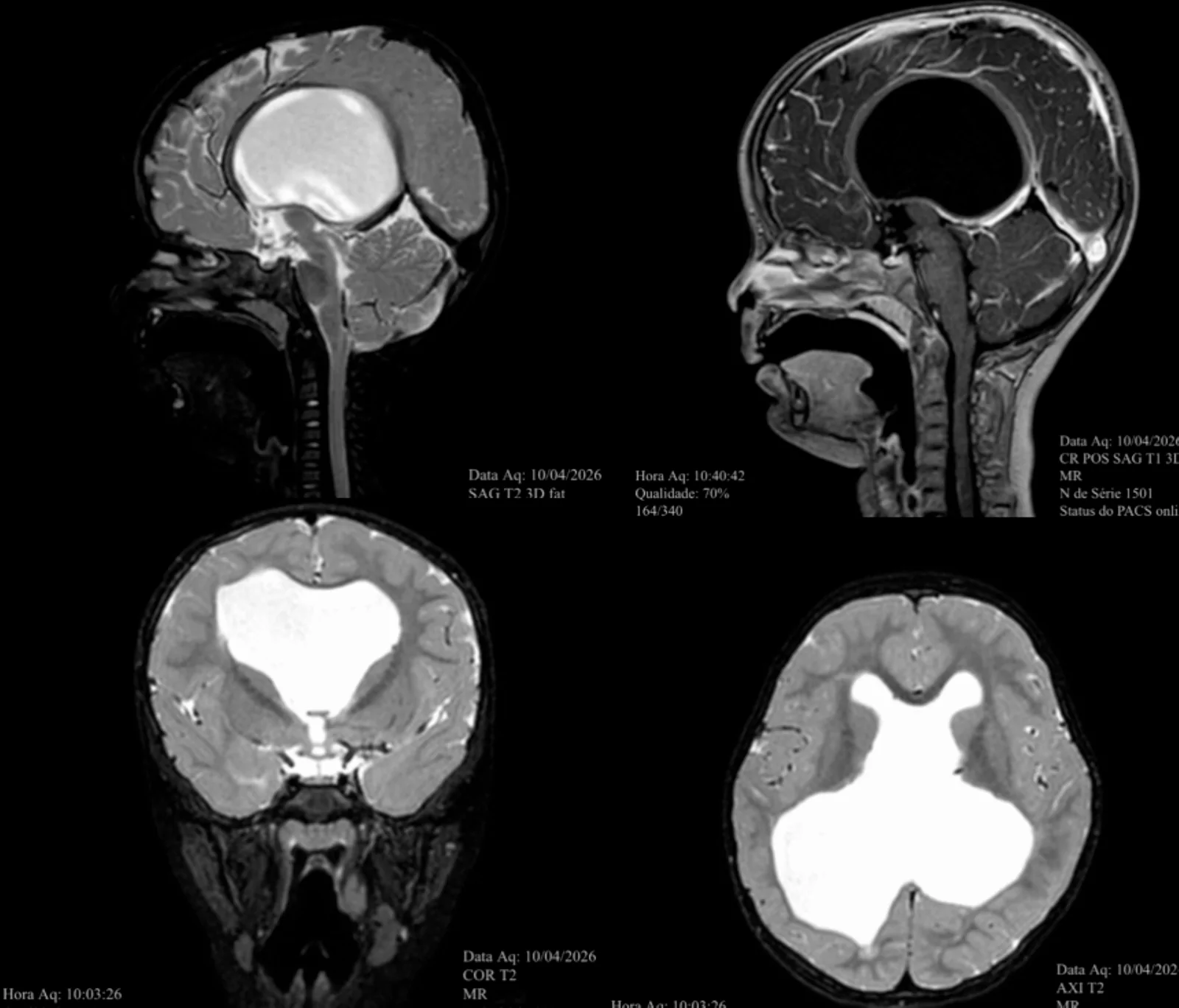
Follow-up MRI demonstrating enlargement of the lateral ventricles, indicating failure of the initial procedure after more than two years

Symptoms showed partial spontaneous resolution, with intermittent recurrence over several days, raising clinical suspicion of intermittent intracranial hypertension secondary to ETV stoma dysfunction. The patient was admitted to the pediatric intensive care unit for monitoring and diagnostic workup. Electroencephalography was unremarkable, effectively ruling out a primary seizure disorder. Neuroimaging demonstrated progressive supratentorial ventricular dilation without transependymal edema, a pattern consistent with ETV stoma occlusion rather than shunt-dependent hydrocephalus (Fig. 4).

Based on the clinical and radiological findings, endoscopic revision was performed. Intraoperative findings confirmed occlusion of the previously created stoma by a membrane at the level of the Liliequist membrane. A new fenestration of the tuber cinereum was performed, together with complete opening of the arachnoid at the Liliequist membrane.

**Fig. 5:**
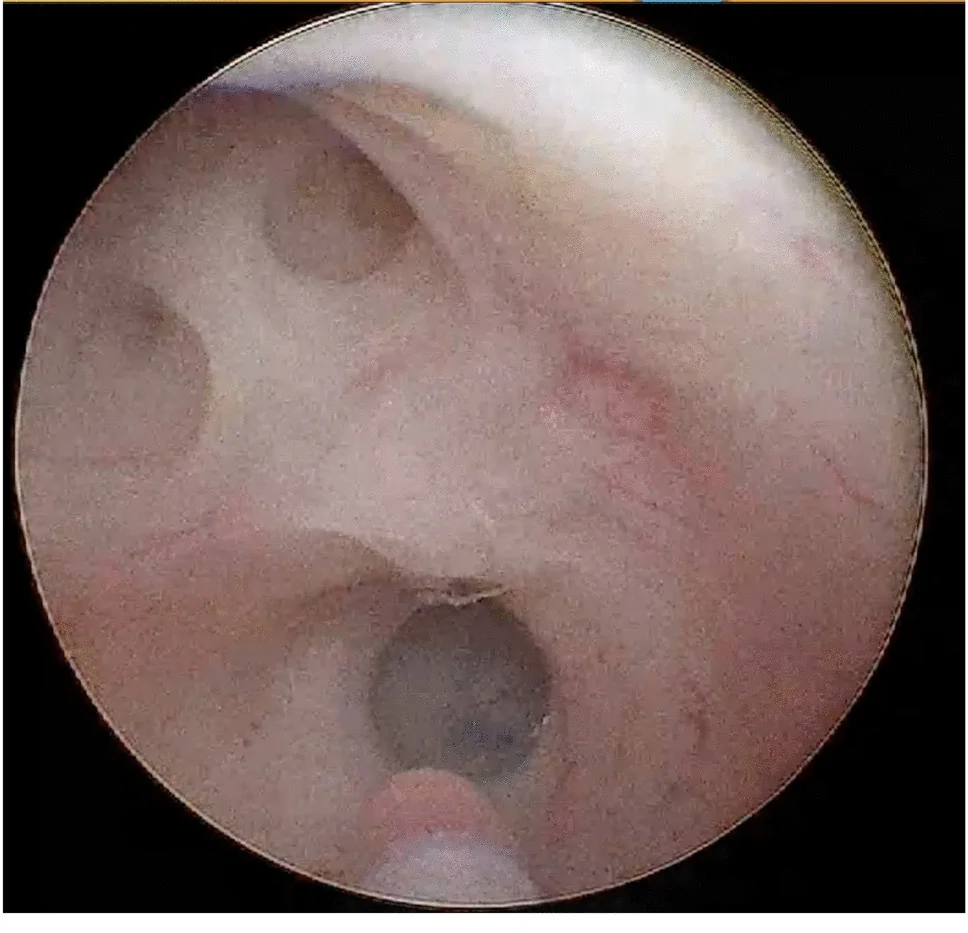
Closure of the third ventricular floor by a neomembrane

**Fig. 6:**
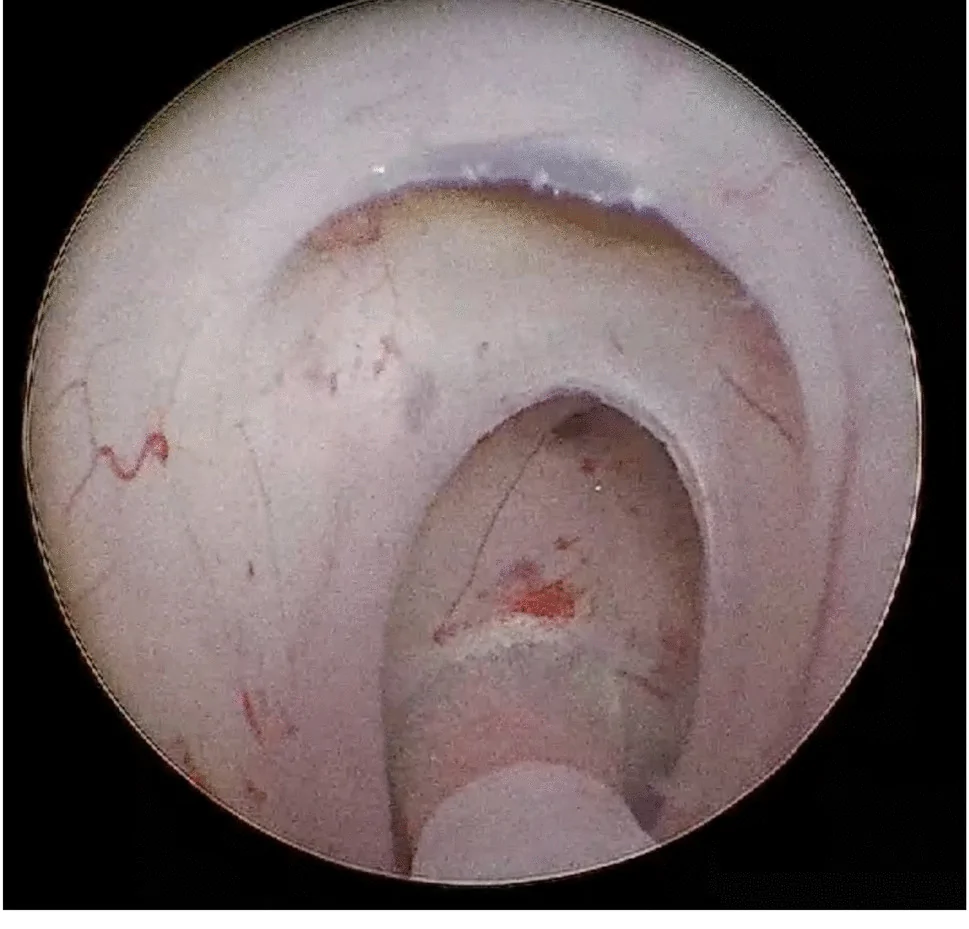
Inflation of a 4 Fr Fogarty balloon during third ventriculostomy

**Fig. 7:**
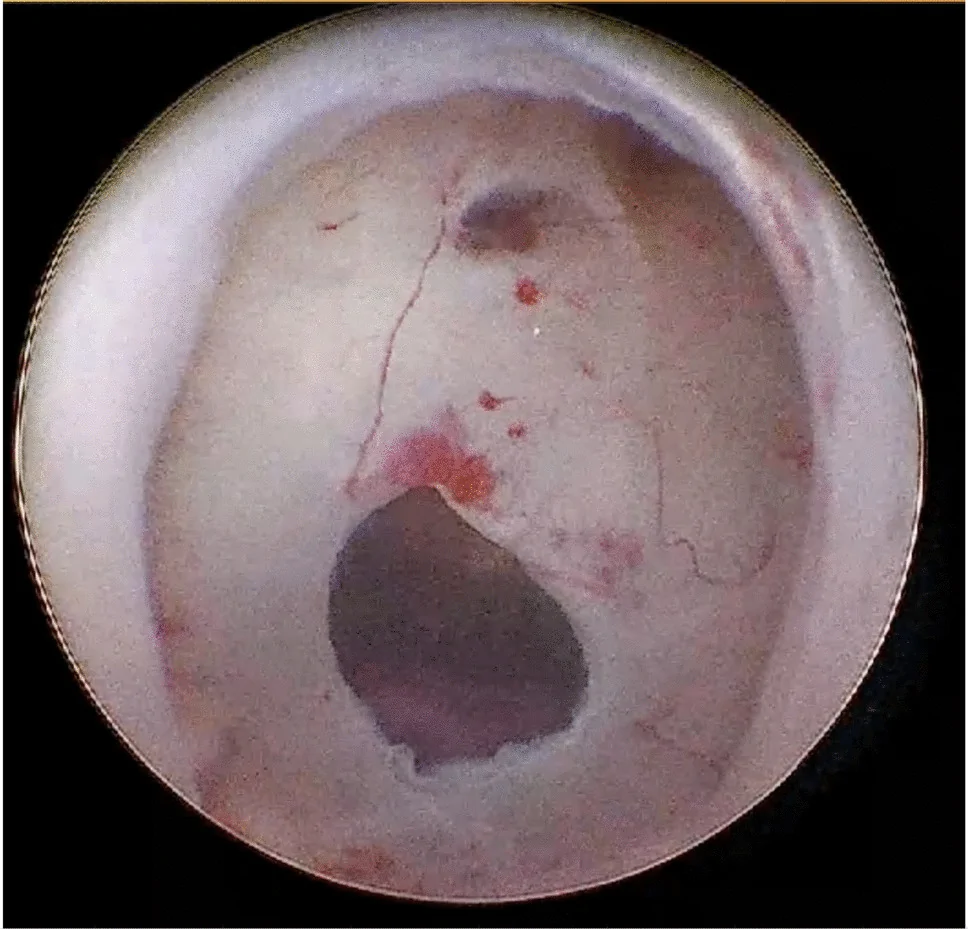
Wide opening in the floor of the third ventricle

The so-called flag sign was observed, indicating adequate membrane mobility and confirming creation of a new, functional stoma. The basilar artery was clearly visualized throughout the procedure, confirming adequate access to the prepontine cistern and allowing safe completion of the fenestration. The procedure was concluded without complications (Figs. 5, 6, and 7).

At the most recent follow-up, 2 years after the endoscopic revision (at approximately 4 years and 5 months of age), the patient remains clinically well. She shows age-appropriate school performance and neuropsychomotor development, with no recurrence of the symptoms that prompted the endoscopic revision. At no point in her clinical course has ventriculoperitoneal shunt placement been required.

## Discussion

This report describes what is possibly the first documented case of endoscopic third ventriculostomy used as the primary surgical treatment of congenital hydrocephalus in a genetically confirmed patient with MPDZ syndrome, performed on the fifth day of life, and documents the successful management of late stoma occlusion by means of endoscopic revision.

The decision to perform ETV was based on the obstructive anatomy identified on fetal MRI: third ventricle atresia associated with diencephalosynapsis and secondary occlusion of the aqueduct of Sylvius. This obstructive mechanism directly supports the indication for ETV, which creates an alternative pathway for CSF circulation by fenestrating the third ventricular floor toward the prepontine cistern, bypassing the site of obstruction.

Case analyses combining pre- and postnatal neuroimaging with fetopathological studies have shown that ventriculomegaly in this condition is consistently obstructive, resulting from partial or complete thalamic fusion leading to third ventricle atresia—a pattern that, notably, does not involve dilation of the third ventricle itself, distinguishing it from classic aqueductal stenosis.

Historically, VPS has been the standard surgical approach in MPDZ cases. A systematic review of published cases suggests that ETV had not previously been reported as primary treatment in liveborn patients with confirmed pathogenic MPDZ variants (Table 1).

**Table 1:** Patients with pathogenic MPDZ variants who survived birth—neurosurgical treatment and ventricular anatomy

Reference	Patient/sex/age	Survived birth	Ventricular anatomy (neuroimaging)	VPS performed?	VPS revision?	ETV/3VT performed?	Outcome/notes
** Al-Dosari et al., 2013**[1]—J Med Genet ·c.628C>T (p.Gln210*), homozygous ·2 Saudi families							
Al-Dosari et al. [1]	F, premature (34 wk) daughter of subject A	Yes	Extreme supratentorial dilation (posterior-predominant). Third ventricle not visualized. Aqueduct without significant dilation. Corpus callosum not identified. Parietal lissencephaly	Yes (day 25)	Not reported	No	Alive at 10 mo; rolling and babbling
Al-Dosari et al. [1]	M, 24 y brother of subject A	Yes	Recent CT: normal V3/V4 after VPS. Multiple catheters in place. Bilateral fronto-basal lissencephalic pattern. Diffuse thinning/reduced volume of corpus callosum	Yes (age 1 y)	Yes—“multiple catheters” on CT → serial revisions	No	Alive; IQ ~ 60; seizures; nystagmus
Al-Dosari et al. [1]	F, 12 y sister of subject A	Yes	CT: catheter in place. Normal V3/V4 post-VPS. Bilateral fronto-parietal cortico-lissencephalic dysplasia. Suggested corpus callosum dysgenesis	Yes (age 1 mo)	Not reported	No	Alive; IQ ~ 80; alternating esotropia
** Saugier-Veber et al., 2017** [20]**—Acta Neuropathol Commun ·Homozygous null mutations· 3 families**							
Saugier-Veber et al. [20]	F, neonate family 2 (sibling)	Yes	Severe ventriculomegaly. Ventricular anatomy details not reported (parents declined sibling autopsy)	Yes (shortly after birth)	Died (before any revision)	No	Died: post-VPS meningoencephalitis
** Shaheen et al., 2017** [22]**—Ann Neurol· Diverse MPDZ variants· Familial congenital hydrocephalus cohort**							
Shaheen et al. [22], case 18	M, 15 mo Kuwaiti ·c.5278G>A	Yes	Enlarged lateral ventricles AND V3, with widened extra-axial spaces. Increased MRI signal in central tegmental tracts and frontal horns	Not reported	Not reported	No	15 mo; iris coloboma; concomitant intrahepatic cholestasis
Shaheen et al. [22], case 16	F, 2.5 y Palestinian· c.4469del	Yes	Dilation of lateral ventricles AND V3. Congenital diaphragmatic hernia. Moderate ASD. Aberrant subclavian artery. Facial asymmetry	Not reported	Not reported	No	2.5 y; motor developmental delay
Shaheen et al. [22], case 17	M, 8 y Scottish–Dutch· c.2230C>T + c.3211C>T	Yes	Minimal lateral ventricle dilation. Enlarged massa intermedia (partial diencephalosynapsis). Bilateral nodular subependymal heterotopia. Small olfactory bulbs	Not reported	Not reported	No	8 y; sensorineural hearing loss; bilateral foveal dysplasia
** Al-Jezawi et al., 2018** [2]**—BMC Med Genet· c.394G>A + c.1744C>G, compound heterozygous· 1 Emirati family**							
Al-Jezawi et al. [2]	M, 9 mo Emirati	Yes	Widened fronto-parietal CSF spaces. Mild lateral ventricle dilation. V3 visible, mildly dilated—non-progressive communicating hydrocephalus. Partial spontaneous resolution confirmed on MRI at 17 mo	Not required	N/A	No	Partial spontaneous resolution (MRI at 17 mo). Normal development
** Rad et al., 2024** [16]**—Clin Genet· Four unrelated families· Expanded phenotypic spectrum**							
Rad et al. [16], family 4	M, 4 y c.4282_4288del, homozygous	Yes	Ventriculomegaly. Absent septum pellucidum. Widened interthalamic adhesion (diencephalosynapsis). Hypoplastic optic nerves/chiasm. Associated septo-optic dysplasia	Not reported	Not reported	No	Bilateral macular coloboma; epilepsy (onset 1 y)
** Stopak et al., 2025** [24]**—J Child Neurol c.2650-1G>A, homozygous ·2 Yemeni siblings**							
Stopak et al. [24]	F, proband alive	Yes	Ventriculomegaly + Dandy–Walker malformation. Subependymal heterotopia. Aortic coarctation. Megalocornea. Surgical treatment of hydrocephalus not specified	Not reported	Not reported	No	Brother died in utero; proband alive, in follow-up
**Cabet and Guibaud et al., 2025 **[3]**—Genes ·Diencephalosynapsis/V3 atresia· 3 families (6 cases, 3 survivors)**							
Cabet & Guibaud et al. [3], P1, family 1	F, 20 mo c.4201-2A>G, homozygous	Yes	Fused thalami. Partial V3 atresia. Mild ventriculomegaly (11 mm). Upstream obstructive pattern. Partially obstructed aqueduct. Normal V4	Not required	N/A	NO (ETV/3VT)—surgical ventriculocisternostomy at 4 mo, distinct from conventional ETV	Mild delay at 20 mo; good surgical response
Cabet and Guibaud et al. [3], P2, family 1 (brother)	M, 4 y 8 mo c.4201-2A>G, homozygous	Yes	Spontaneous postnatal resolution. Partial V3 atresia. Partially fused thalami. Normal V4	Not required	N/A	No	Normal neurodevelopment; mild attentional disturbance
Cabet and Guibaud et al. [3], P4, family 2	M, 13 mo c.5191A>T + c.4807 + 1G>A	Yes	Fused thalami. Partial V3 atresia. Mild, stable ventriculomegaly. Periventricular nodular heterotopia. Normal V4	Not required	N/A	No	Normal neurodevelopment at 13 mo; concomitant cardiopathy
**Cavalheiro et al. (UNIFESP/2026) ·**							
Present case	F (prenatal dx, born 36 + 1 wk)	Yes	Bilateral ventriculomegaly; hypoplastic septum pellucidum/corpus callosum; diencephalosynapsis; third ventricle atresia; secondary aqueductal occlusion	No	N/A	First reported case—primary ETV (day 5 of life); endoscopic revision at 2 y 5 mo for late stoma occlusion	Favorable neurodevelopment at 4 y 5 mo follow-up; no VPS dependency

Systematic literature review. Only liveborn cases are included; terminated pregnancies and stillbirths were excluded

*VPS *ventriculoperitoneal shunt, *ETV/3VT* endoscopic third ventriculostomy, *V3* third ventricle, *V4* fourth ventricle, *N/A* not applicable

ETV in neonates and young infants presents recognized challenges, including a higher rate of stoma closure related to cranial compliance, reduced arachnoid pulsatility, and greater thickness of the third ventricular floor compared with older children and adults.

In the presented case, several factors argued in favor of proceeding with an endoscopic approach rather than primary shunting. The hydrocephalus was clearly obstructive, related to third ventricle atresia and diencephalosynapsis without any history of intraventricular hemorrhage or central nervous system infection. Intraoperative findings additionally demonstrated favorable ventricular anatomy for an endoscopic approach, including a thin, bulging third ventricular floor, a feature that has itself been proposed as a predictor of ETV success in infants independent of age-based scoring systems [27].

Furthermore, systematic reviews and meta-analyses have demonstrated that ETV remains a feasible and comparably safe treatment option for selected infants with non-communicating hydrocephalus, including those younger than 6 months [25, 29]. In the largest dedicated systematic review of ETV outcomes in children 1 year of age or younger, congenital aqueductal stenosis was the most common underlying etiology, and overall ETV success rates remained above 50% even though younger age was associated with a lower probability of success compared with older infants [29].

Similarly, a focused meta-analysis of ETV versus ventriculoperitoneal shunting (VPS) specifically in aqueductal stenosis found that, although ETV carried a higher, non-statistically significant failure rate compared with shunting in infants younger than 24 months, functional and long-term outcomes did not differ significantly between the two techniques [18]. Broader comparative literature further supports ETV as a reasonable first-line strategy whenever an obstructive mechanism can be demonstrated, given its lower infection-related morbidity relative to VPS [25]. Taken together, these data support the rationale for offering ETV as primary treatment in this neonate, based on the obstructive, non-infectious, non-hemorrhagic nature of her hydrocephalus and on favorable ventricular anatomy, rather than on age alone.

Late ETV failure, defined as stoma dysfunction occurring more than 6 months after the initial procedure, is a recognized entity in the literature, occurring predominantly within the first 5 years after surgery. The clinical presentation in this case was notable for its intermittent, subacute character, with episodes of somnolence, bradycardia, pallor, and vomiting showing partial spontaneous resolution, suggesting intermittent obstruction rather than acute, complete failure.

This pattern underscores the importance of continued clinical vigilance, even in patients who remain asymptomatic for long periods after an initially successful ETV. Endoscopic revision with refenestration of the tuber cinereum and complete opening of the Liliequist membrane proved effective. Observation of the “flag sign” and clear visualization of the basilar artery confirmed the adequacy of the new stoma.

This case reinforces the multisystemic nature of MPDZ syndrome beyond isolated hydrocephalus. The coexistence of vesicoureteral reflux, pyelocaliceal duplication, and a patent foramen ovale requiring surgical correction is consistent with the recently described expansion of the phenotypic spectrum. The multisystem involvement observed underscores the importance of comprehensive systemic evaluation at diagnosis, including cardiac, renal, and ophthalmologic assessment.

## Conclusion

We report the first documented case of endoscopic third ventriculostomy used as primary treatment for MPDZ-related obstructive hydrocephalus, performed at 5 days of life, with successful management of late occlusion of the Liliequist membrane by endoscopic revision at 2 years and 5 months of age.

The favorable outcomes observed in this case—successful primary ETV without shunt dependency, successful endoscopic revision, and appropriate neurodevelopment at 4 years and 5 months of age—provide proof of concept that ETV is not only mechanistically justified but also clinically effective in MPDZ-related hydrocephalus. Avoidance of VPS dependency and its associated chronic complications represents a clinically meaningful benefit.

Nevertheless, MPDZ syndrome shows considerable phenotypic variability, ranging from mild ventriculomegaly requiring no treatment to severe hydrocephalus associated with early neonatal death. Although the obstructive mechanism provides a general rationale for ETV, patient selection should remain individualized, taking into account age, degree of ventriculomegaly, thickness of the third ventricular floor, and associated structural anomalies.

We advocate for prospective reporting of ETV outcomes in future cases of MPDZ syndrome in order to build a robust evidence base for this therapeutic approach.

## Data Availability

No datasets were generated or analysed during the current study.
